# Single cell transcriptional zonation of human psoriasis skin identifies an alternative immunoregulatory axis conducted by skin resident cells

**DOI:** 10.1038/s41419-021-03724-6

**Published:** 2021-05-06

**Authors:** Yuge Gao, Xinyu Yao, Yumeng Zhai, Li Li, Huini Li, Xianqi Sun, Pei Yu, Tiankuo Xue, Yuzhen Li, Yizhou Hu

**Affiliations:** 1 Department of Dermatology, The Second Affiliated Hospital of Harbin Medical University, No.246 Xuefu Road, 150001 Harbin, China; 2 Institute of Biotechnology, HILIFE Unit, University of Helsinki, 00014 Helsinki, Finland; 3 Department of Dermatology, Peking University First Hospital, No.8 XiShiKu Street, 100034 Beijing, China; 4 Department of Oncology, The Second Affiliated Hospital of Harbin Medical University, No.246 Xuefu Road, 150001 Harbin, China; 5 Division of Molecular Neurobiology, Department of Medical Biochemistry and Biophysics, Karolinska Institutet, Solnavägen 9, 17165 Solna, Stockholm Sweden

**Keywords:** Psoriasis, Psoriasis

## Abstract

Psoriasis is the most common skin disease in adults. Current experimental and clinical evidences suggested the infiltrating immune cells could target local skin cells and thus induce psoriatic phenotype. However, recent studies indicated the existence of a potential feedback signaling loop from local resident skin cells to infiltrating immune cells. Here, we deconstructed the full-thickness human skins of both healthy donors and patients with psoriasis vulgaris at single cell transcriptional level, and further built a neural-network classifier to evaluate the evolutional conservation of skin cell types between mouse and human. Last, we systematically evaluated the intrinsic and intercellular molecular alterations of each cell type between healthy and psoriatic skin. Cross-checking with psoriasis susceptibility gene loci, cell-type based differential expression, and ligand-receptor communication revealed that the resident psoriatic skin cells including mesenchymal and epidermis cell types, which specifically harbored the target genes of psoriasis susceptibility loci, intensively evoked the expression of major histocompatibility complex (MHC) genes, upregulated interferon (INF), tumor necrosis factor (TNF) signalling and increased cytokine gene expression for primarily aiming the neighboring dendritic cells in psoriasis. The comprehensive exploration and pathological observation of psoriasis patient biopsies proposed an uncovered immunoregulatory axis from skin local resident cells to immune cells, thus provided a novel insight for psoriasis treatment. In addition, we published a user-friendly website to exhibit the transcriptional change of each cell type between healthy and psoriatic human skin.

## Introduction

Psoriasis is the most common chronic, immune-mediated disorder skin disease in adults, with an estimated global prevalence of 2–3%^1^, of which 90% is psoriasis vulgaris^2^. The pathophysiology of psoriasis vulgaris is characterized by abnormal keratinocyte proliferation and immune cell infiltration in the dermis and epidermis, further affect the overall innate and adaptive immune systems^3^. Therefore, psoriasis vulgaris and its comorbidities have caused serious physical and mental burden on patients all over the world^2,4–6^.

Even though psoriasis is one of the most studied dermatological diseases, its pathogenesis is still not completely elucidated. Accumulating cellular and molecular regulators have been found to be pivotal during the pathogenesis of psoriasis vulgaris. Previous experimental and clinical evidences have established the inflammatory roadmap from infiltrating immune cells including neutrophils, T cells, dendritic cells (DCs) to skin resident cells, especially the keratinocytes^2,3,7^. Most of these circuits are directly or indirectly mediated by chemokines and cytokines. Current knowledge indicated that multiple factors could induce the activation of myeloid dendritic cells (mDCs) and the production of interleukin (IL)-23, which in turn stimulated the major T cell subsets to secrete IL-17 (ref. ^8,9^). This pathogenic model of psoriasis vulgaris based on the IL-23/IL-17 axis is widely accepted^8,10,11^. Likewise, both IL-17 and IL-23 inhibitors have shown good efficacy and safety in the systemic treatment of psoriasis. However, these inhibitors could not completely alleviate the disease^12^, suggesting alternative regulatory events of psoriasis beyond IL-23/IL-17 signaling. Recent studies revealed a few molecular feedback events occurred from the skin resident cells^13,14^, indicating a potential indirect immune regulatory axis driven by the resident cells. However, previous studies mostly either focused on one solitary signaling axis or profiled the genetic alterations of human psoriasis skin at the bulk tissue level^15–17^, which led to the obscuration of the overall contribution of psoriasis-related genetic characteristics to specific cell type. Therefore, urgently exploration is needed to systematically study the inherent transcriptional changes and intercellular molecular communication of each specific cell type in human psoriasis full-thickness skin.

In this study, we used single-cell RNA sequencing (scRNAseq) to deconvolute a high-quality transcriptional landscape of human epidermal and dermal skin cells from both healthy donors and patients with psoriasis vulgaris. Through the combination of neural-network learning, multilayer analysis of genome/transcriptome/ligand–receptor and pathological investigation of patient biopsies, we further revealed the evolutionally conserved skin resident cells, including both epidermal keratinocytes and dermal mesenchymal cells, could self-transform into immune active status by evoking MHC genes during psoriasis vulgaris, and further nutrient immune cells via upregulating cytokine genes and extracellular matrix related genes.

Last, we built a user-friendly website for exploring the gene expression of normal/psoriasis human skin cells: https://yzstudio.one/skin-psoriasis-atlas.

## Result

### Single cell transcriptional profiling of the full-thickness healthy and psoriatic skin

**Fig. 1:**
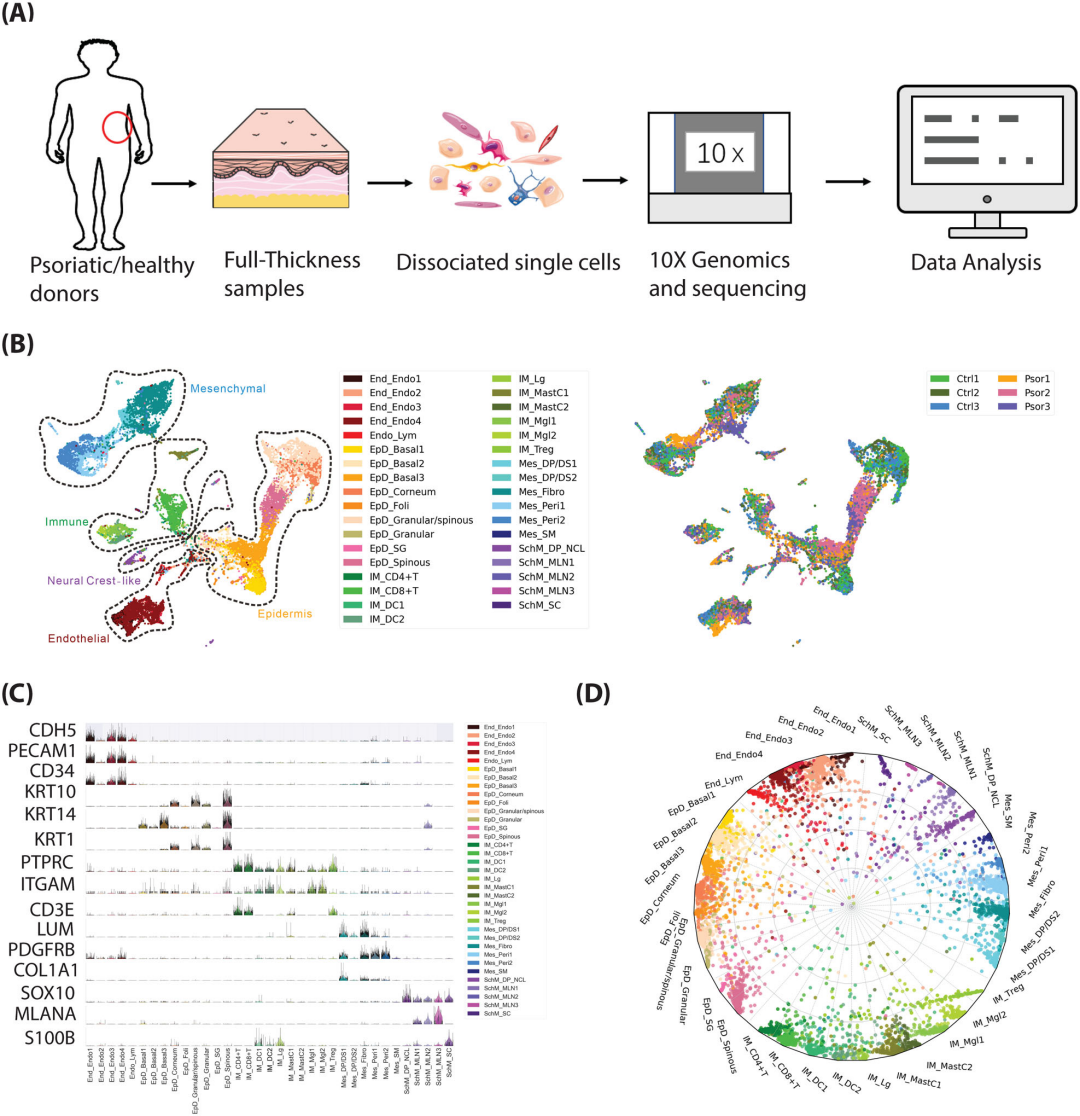
Clustering, marker genes and cell type assignment of human full-thickness skin scRNAseq from healthy and psoriatic donors. **A** Schematic view of the workflow. **B** Left, human full-thickness skin cell types. Visualization using UMAP of skin cells, each point represents one cell. Color coding based on unique cell clusters, as shown at right beside. Right, UMAP plot was folked from left side and colored by samples. Ctrl 1–3, samples from healthy donors; Psor 1–3, samples from patients with psoriasis vulgaris. **C** Expression of selected top significant marker genes for five main cell type clusters; relative expression of each gene in each cluster was visualized as violin plot, and the raw counts of each gene were visualized as sparkline plot in that violin plot. **D** Radar plot visualization of cell-type probabilistic scores of human full-thickness skin cells in relation to our defined cell types. Each dot represented one cell. Color coding based on unique cell clusters. The position of each dot indicated the cell-type score between that cell and the training (defined) cell types which were indicated outside of each bend in the radar wheel. Almost all cells were correctly assigned to their defined cell types.

To systematically and comprehensively address the complexity and potential pathological change of cell types in both dermis and epidermis of patients with psoriasis vulgaris, single cells from full-thickness skins were collected from the lesion region of three patients (two male and one female) and the similar region of three healthy donors (two male and one female). All tissues were subsequently profiled with single cell RNA sequencing (Fig. 1A; SI Table 1). After enzymatically and mechanically disrupting the tissue, single cell suspension (viability >90%) was subjected to scRNAseq. 24,259 single cells were passed the initial quality control with the median detected UMI reads of 8858 and median detected protein-coding genes of 2747, mitochondrial gene ratio <5%. And the doublets (estimated ratio ~3.3%) were removed (SI Fig. 1A-D). In addition, we observed one patient harbored HLA-C*06:02, a primary susceptibility allele for psoriasis^18,19^ (SI Fig. 1E).

All 24,259 cells were further clustered to five main clusters and one erythrocyte cluster (HBA1^+^ HBB^+^ HBA2^+^ cells). After excluding erythrocyte cluster (25 cells), the rest five main clusters of 24,234 cells were further clustered into 35 sub-cell types and visualized in a uniform manifold approximation and projection (UMAP) plot (Fig. 1B, left). Each cell-type contained cells from all investigated donors as visualized in (Fig. 1B, right), but cell-type composition changes of psoriatic and healthy cells varied among some cell types, as described in (Fig. 3A, B). After comparing well known markers with the most representative expressed gene markers of each cluster, five main clusters were defined as follows: endothelial (End/Endo), majorly expressing CDH5, PECAM1, CD34; epidermis (EpD), majorly expressing KRT10, KRT14, KRT1; immune (IM), majorly expressing PTPRC (CD45), ITGAM, CD3E; mesenchymal (Mes), majorly expressing PDGFRB, LUM, COL1A1; neural crest-like (Schwann/Melanocyte-like, SchM), majorly expressing SOX10, MLANA, S100B (Fig. 1C).

As the most abundant skin cell type, EpD accounted for 37.96% (9201 cells) of all skin cells, which could be divided into nine different clusters, including three basal cell types (EpD_Basal1–3) majorly expressing basal epithelium markers KRT5, KRT14, ITGA6, and ITGB1; four epithelial differentiated cell types (EpD_Spinous, EpD_Granualr/spinous, EpD_Granular, EpD_Corneum) majorly recognized by epithelial differentiation marker KRT10, KRT1, and GRHL1 (ref. ^20–23^); sebaceous gland (EpD_SG) expressing APOC1, ACSL5, ABCC3 (ref. ^24^); follicles (EpD_Foli) expressing CD200, SOX9, KRT19 (ref. ^25,26^). End/Endo constituted 15.69% (3802 cells) of the full-thickness skin cells, was identified by its archetypal marker PECAM1, which was represented by four endothelial cell types (End_Endo1–4) and a lymphatic endothelial cell type (Endo_lym)^27^. In total, 7642 mesenchymal cells counted for 31.53% of the full-thickness skin cells were represented by six clusters included fibroblast (Mes_Fibro), 2 fibroblast/pericytes-like cell types (Mes_Peri1–2), two dermal sheath/dermal papilla cell types (Mes_DS/DP_1–2), and smooth muscle-like (Mes_SM). We detected 2983 immune cells, 12.3% of the total cells, and divided them into 10 clusters, namely three T cell types (IM_CD4+, IM_CD8+, IM_Treg), two dendritic cell types (IM_DC1–2), Langerhans (IM_Lg), two mast cells (IM_MastC1–2), and two macrophage-like cells (IM_Mgl1–2). We detected 606 SOX10+ cells as SchM neural crest-related cells, occupying 2.5% of all skin cells, including three melanocyte clusters (SchM_MLN1–3), a dermal papilla cell/neural crest-like cluster (SchM_DP_NCL), and one Schwann cell cluster (SchM_SC). Each cluster had unique transcriptional markers (SI Table 2a), and was in line with previous known markers in that type of cells (SI Table 2b)^28–30^, selected marker genes were visualized in (SI Fig. 1E). To further validate the accuracy of our classification, we applied cross-validation driven by a vanilla neural-network. The classifier successfully assigned each cell into their defined cluster (Fig. 1D) with an accuracy near 80% (SI Fig. 1F, G). Randomized expression matrix was used as negative control, and in the same model, none of randomized cells significantly assigned to the right cluster (SI Fig. 1H), suggesting the robustness of our classification. The expression of all protein-coding gene of each cluster was visualized as violin-plot and UMAP plot in the webpage: https://yzstudio.one/skin-psoriasis-atlas.

### The anatomical location and evolutional conversation of human skin cell type

**Fig. 2:**
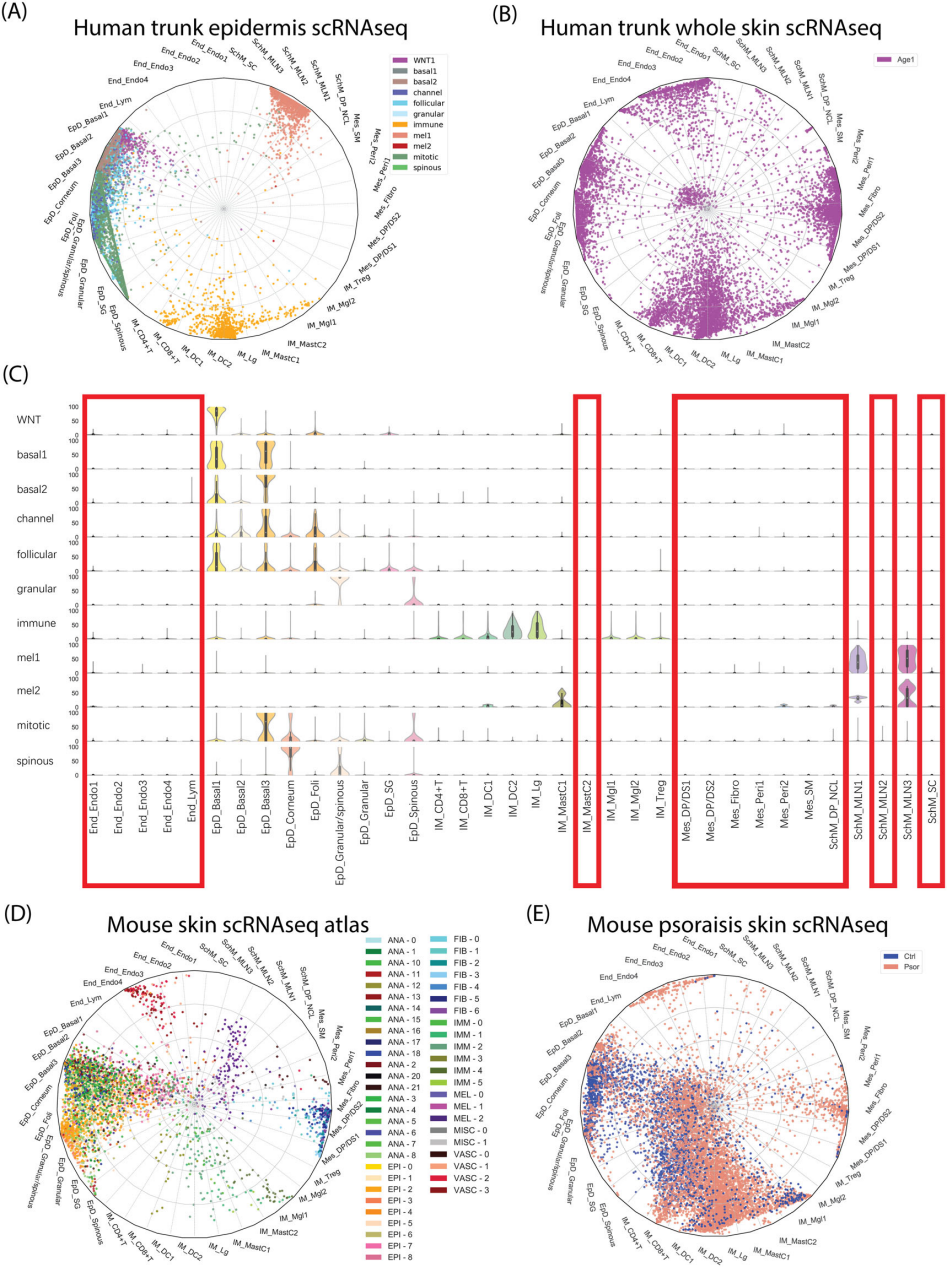
Prediction of the anatomical location and evolutional conversation of human skin cell type. **A** Radar plot visualization of cell-type probabilistic scores of published human trunk epidermis skin cells in relation to our defined cell types. **B** Radar plot visualization of cell-type probabilistic scores of published human full-thickness skin cells in relation to our defined cell types. **C** Violin plot visualization of cell-type probabilistic scores of published human trunk epidermal skin cells in relation to our defined cell types. *X-*axis represented our defined cell types, *y-*axis represented defined cell types from original study. Red frame indicated low similar (absent) cell types of our defined cell types in epidermal skin cell types. **D** Radar plot visualization of cell-type probabilistic scores of published mouse skin atlas cells in relation to our defined human skin cell types. **E** Radar plot visualization of cell-type probabilistic scores of published mice healthy or psoriatic skin cells in relation to our defined human skin cell types. For all radar plots, each dot represented one cell. Color coding based on their original defined cell clusters or disease condition. The position of each dot indicated the cell-type score between that cell and the training (defined) cell types which were indicated outside of each bend in the radar wheel.

To explore the anatomical regions of our defined cell types, we compared our data with published human skin scRNAseq data only from epidermis layer^31^ or from full-thickness skin^32^. To exert the cell-type comparison at single cell resolution, we applied previously built neural-network classifier (Fig. 1D), which was used the trained knowledge-supervised cell-type as machine learning model to evaluate the weighted gene-pattern relations and then to systematically quantify the probabilistic similarity of each predicted cell against our defined cell types. Therefore, the cell-to-cell type similarity did not rely on a few marker genes, but the comprehensively weighted gene patterns, and thus significantly enhanced the evaluation accuracy. We successfully assigned epidermis skin cells (Fig. 2A) and full-thickness skin cells (Fig. 2B) into our clusters, as visualized in radar plot. Overall, most of the predicted full-thickness skin cells were assigned to our defined cell types (Fig. 2B), and all our defined cell types contained the predicted full-thickness skin cells, indicating that our defined cell types could correctly classify and represent the full-thickness skin cells from other published data. In contrast, all epidermal skin cells were only assigned to some of our defined cell types, including all epidermis clusters, most immune cell types except IM_MastC2, and two types of melanocytes (SchM_MLN1, SchM_MLN3). Compared with the assignment of full-thickness skin data, we confidently identified the cell types specifically at skin dermis or the junction region of epidermis/dermis, including all endothelial cells, all mesenchymal cells, IM_MastC2 mast cells, a type of melanocyte (SchM_MLN2), dermal papilla cell/neural crest-like cluster (SchM_DP_NCL), and Schwann cells (SchM_SC), as indicated with red frame in (Fig. 2C). This finding was in line with previous studies that most blood vessel cells and mesenchymal cells located in the skin dermis^29,32^, and most of the human melanocytes located among keratinocytes in the epidermis layer or junction region^33^, while Schwann precursor cells had been collected from adult human dermis^34,35^.

Next, we evaluated the evolutional conservation of human and mouse skin cell types. The same neural-network classifier was applied to compare our human cell types with published mouse scRNAseq data from mouse skin^30^ or psoriatic mouse skin^36^. Among five main cell types, three main types including epidermis, endothelial, and mesenchymal were relatively evolutional conserved with the minor inconsistence that mouse skin harbored much less epidermis granular cells (EpD_Granular), epidermis hair follicle cells (EpD_Foli), epidermis sebaceous gland cells (EpD_SG), despite the different experimental protocols and capture efficiencies (Fig. 2D; SI Fig. 2). We observed in this dataset that psoriatic mouse skin had significant high ratio of different types of immune cells than normal mouse skin (Fig. 2E), in line with the observation of infiltrating immune cells in skin region during psoriasis. Moreover, all the immune cell types were also similar to human skin except Treg cells. In contrast to the four main types above, most SOX10^+^ cell types (SchM main types) exhibited significantly evolutional insistence, although the major trend of similarity was observed. None of neural crest-like cells of mouse skin was exactly assigned to human SOX10^+^ cell types, except a few of psoriatic mouse skin cells were assigned to human melanocytes (SchM_MLN3) and human Schwann cells (SchM_SC).

### Skin resident cell types exhibited immune regulatory ability

**Fig. 3:**
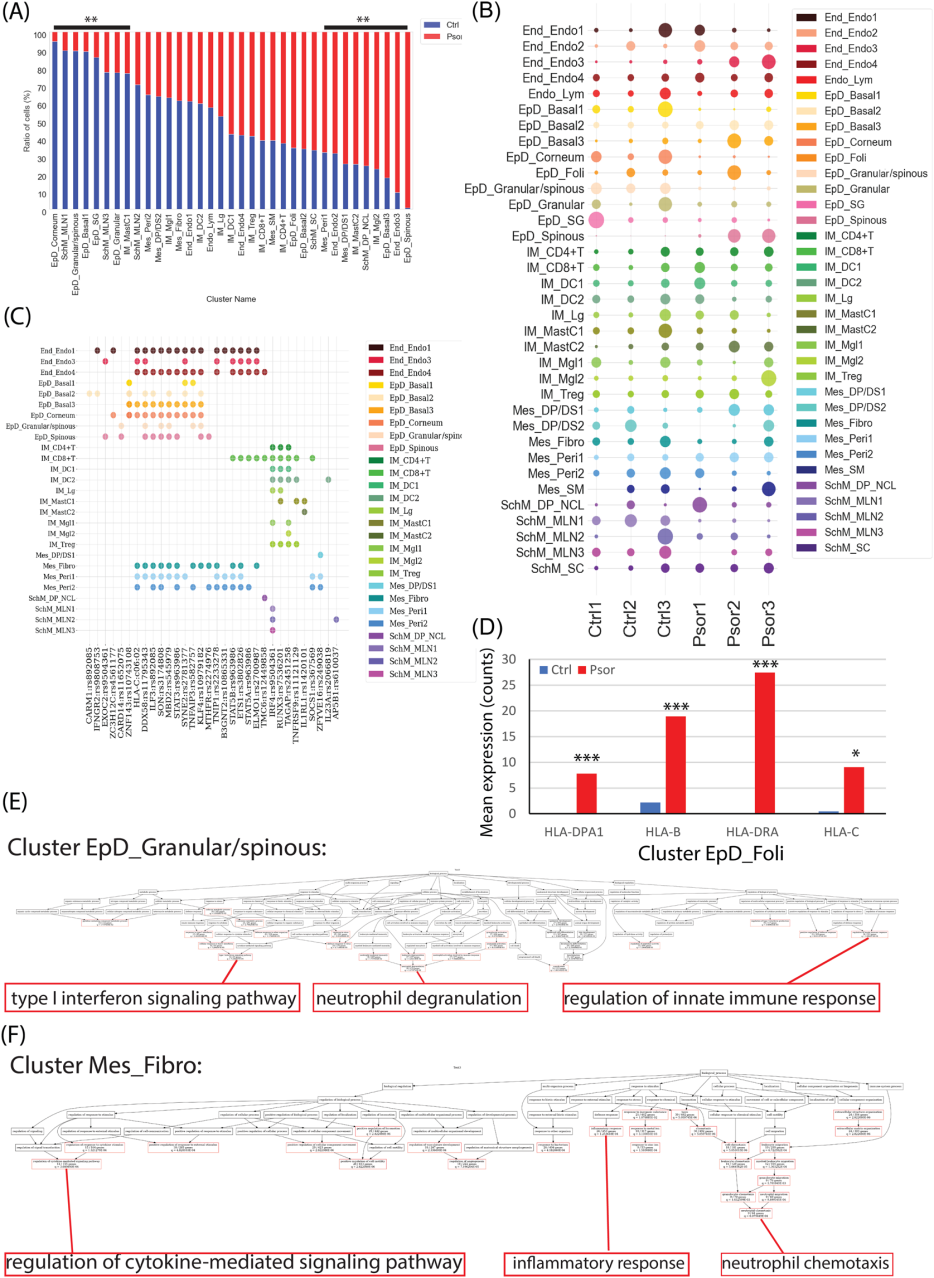
The difference of cellular composition and cell-type-based transcription in psoriasis. **A** Percentage bar plot of cell type composition in healthy and psoriatic skin, *y-*axis represented the percent of total cell number of that cell type. Red color represented psoriatic skin and blue color represented healthy control skin. Significant changes (FDR < 0.01) were labeled as ** on the top. **B** Dot plot of cell number distribution of each cell type in each healthy and psoriasis donor. Dot size represented the cell number percent of that donor (*x-*axis) in that cell type (*y-*axis). Color indicated the cell types. **C** Dot plot of the significant existence of each susceptibility gene loci (*x-*axis) in each cell type (*y-*axis). Color indicated each cell type. **D** Bar plot of the raw counted of four selected HLA genes in cell type EpD_Foli. Red color represented psoriatic skin and blue color represents healthy control skin. *Y-*axis represents the mean value of the expression raw counts. ***, FDR *p* < 0.001; *, *p* < 0.05. **E**, **F** GO-bioprocess enrichment plot of all psoriasis upregulated genes in cell type EpD_Granular/spinous (E) and Mes_Fibro (F). Red frame highlighted the significant enriched GO terms and the terminally enriched GO-bioprocess were enlarged at the bottom of each plot.

We further analyzed the cell-type distribution of different individuals, including 11,417 cells from three healthy donors and 12,817 cells from three donors with psoriasis, and observed the changes of cell-type composition between psoriasis and the health. Statistical analysis revealed that EpD_Corneum, SchM_MLN1, EpD_Granular/spinous, EpD_Basal1, EpD_SG, SchM_MLN3, EpD_Granular, IM_MastC1 cells showed a significant decreasing in psoriasis, while EpD_Spinous, End_Endo3, EpD_Basal3, IM_Mgl2, SchM_DP_NCL, IM_MastC2, Mes_DP/DS1, End_Endo2, Mes_Peri1 cells increased significantly (Fig. 3A, B).

The cell-type composition changes largely reflected cell viability (autonomous) and motility (non-autonomous), and indicated the cell-based functional shift in this region. We further zoomed into the molecular changes of each cell type during psoriasis, which included three aspects: first, cell type assignment of psoriasis susceptibility gene loci; second, the differentially expressed genes among each cell type induced by psoriasis; last, the reorganized ligand–receptor interaction among each cell type.

First, we collected 45 experimentally identified psoriasis susceptibility gene loci and their target genes, including the primary susceptibility gene loci and HLA-C*06:02 (SI Table 3)^37,38^. The cell types significantly expressing these genes were the most vulnerable cellular targets of the psoriasis genetic predisposition. After cross-checking of both 45 susceptibility gene loci and our cell-type marker genes. We identified 32 common psoriasis susceptibility gene loci, which were differentially distributed in 35 cell types of skin (Fig. 3C), the gene expression of each cell type could be searched in our webpage: https://yzstudio.one/skin-psoriasis-atlas. Most of these loci were related to immune regulation, especially IFN and TNF signaling, such as IFNGR2, IL23A, STAT3, STAT5, TNFAIP3, and so on. Interestingly, 24 out of 32 target genes were highly or shared expressed in local resident skin cells, especially in epidermal cells and mesenchymal cells, including CARM1, IFNGR2, EXOC2, ZC3H12C, CARD14, ZNF143, DDX58, HLA-C, ILF3, SON, MBD2, STAT3, SYNE2, TNFAIP3, KLF4, MTHFR, TNIP1, B3GNT2, STAT5B, ETS1, STAT5A, ELMO1, SOCS1, ZFYVE16. This result indicated that the psoriasis genetic predisposition could not only target commonly known immune cells, but also perturbate local resident skin cells including both epidermis cells and mesenchymal cells, with endorsing immuno-regulatory potential.

Next, we compared the differential gene expression of each cell type between healthy and psoriatic human skin. Statistical analysis enriched both upregulated and downregulated genes of each cell type in psoriatic human skin. Surprisingly, some local resident skin cell types significantly upregulated MHC complex molecules in psoriatic condition: epidermal cell type EpD_Basal2, EpD_Basal3, EpD_Foli highly expressed MHC-II molecules such as HLA-DRA, HLA-DMA, HLA-C; mesenchymal cell types Mes_DP/DS1, Mes_Fibro, Mes_Peri1, Mes_Peri2 highly expressed both MHC-I and MHC-II molecules such as HLA-A, HLA-B, HLA-C, HLA-DRA. Besides, dermal papilla cell/neural crest-like cells (SchM_DP_NCL) and Schwann cells (SchM_SC) highly expressed MHC-I molecules such as HLA-A, HLA-B, HLA-C. An example of the upregulated expression of MHC complex genes in EpD_Foli was shown as bar chart in (Fig. 3D) and all differential expression data was listed in (SI Table 4) and searchable in our webpage: https://yzstudio.one/skin-psoriasis-atlas.

Furthermore, gene ontology enrichment (GO) analysis of the significant upregulated genes of epidermis and mesenchymal cell types revealed two types of main GO bioprocesses: First, immune-responses including interferon signaling, neutrophil modulation, cytokine and chemokine production, and so on; Second, the regulations of cell cycle and cell apoptosis, which were majorly activated in epidermal basal cells (EpD_Basal1–3). Thus, the upregulated genes of local resident skin cells mainly contributed to immune modulation during psoriasis. Representative GO-bioprocess enrichment of cell type EpD_Granualar/spinous was shown in (Fig. 3E, F) and full enrichment figures and lists of each cell type were in (SI Fig. 3; SI Table 5).

### Ligand–receptor analysis revealed the regulatory potential from resident epidermal/mesenchymal cells to dendritic cells during psoriasis

**Fig. 4:**
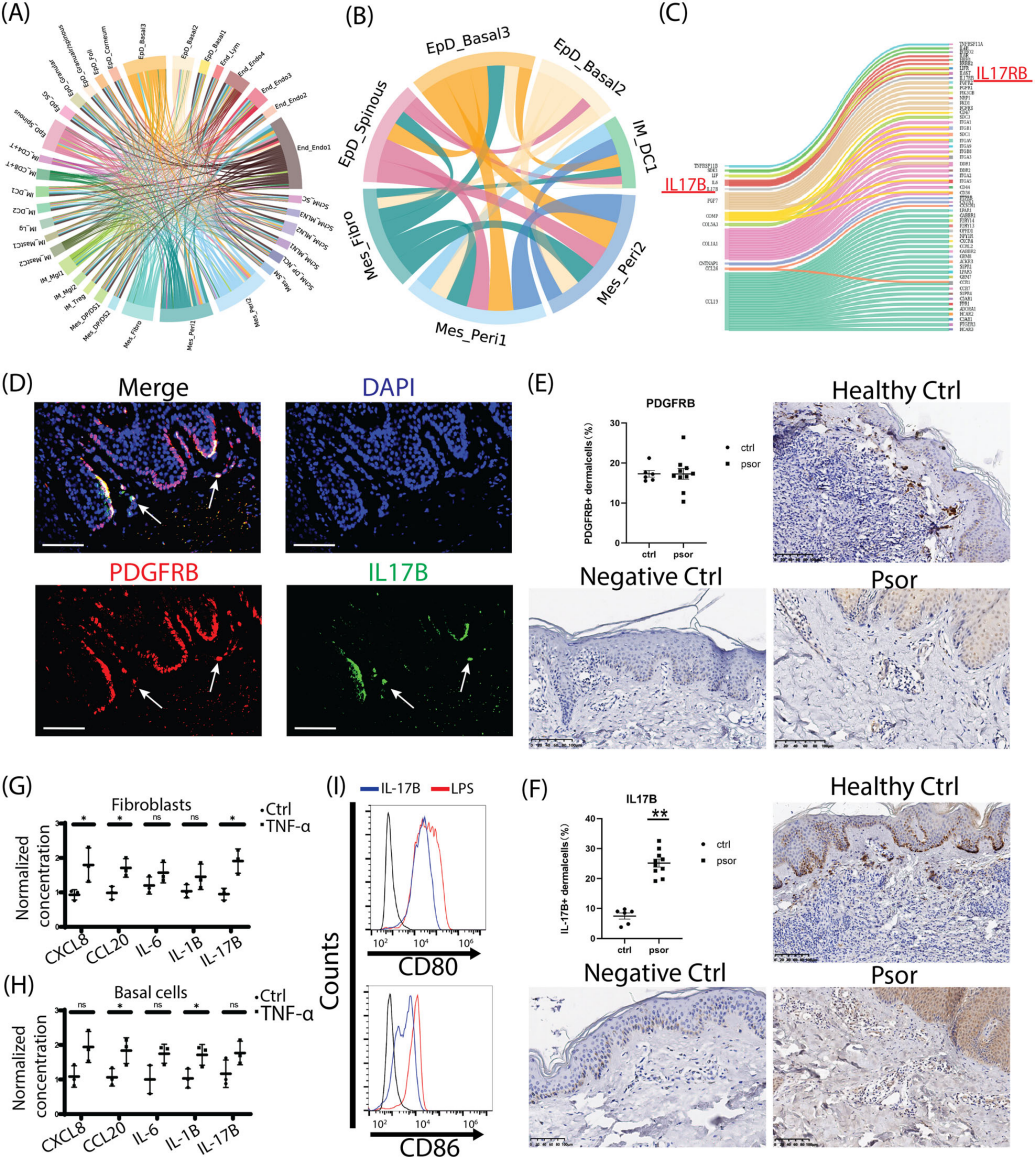
Molecular interactions among cell types and the regulatory potentiality from epidermal/mesenchymal cells to dendritic cells during psoriasis. **A** Chord plot visualization of the ligand-receptor interactions among cell types. The defined cell types were indicated outside of each bend. Arc line between each cell types represented the ligand to receptor interactions, and the color indicated the ligand expressed cell types. **B** Chord plot visualization of the ligand-receptor interactions between top scored resident cell types and type 1 DC. The defined cell types were indicated outside of each bend. Arc line between each cell types represented the ligand to receptor interactions, and the colors indicated the ligand expressed cell types. **C** Sankey plot visualization of the interactions from the ligand of mesenchymal type cells to the receptors of type 1 DCs. The IL-17B/IL-17RB pair was highlighted as red. **D** Immunofluorescence staining of the epidermis/dermis junction of human skins, PDGFRB protein was stained as red, IL-17B as green, and DAPI as blue, the cellular co-expressions of PDGFRB and IL-17B were observed at the dermis layer close to the junction (pointed by white arrow) and at the layer junction. Scale bar, 100 µM. **E**, **F** Immunohistochemistry staining and quantification of PDGFRB (**E**) and IL-17B (**F**) in healthy and psoriatic skins. Host IgG of primary antibodies were used as negative control. In dermis layer, the average PDGFRB protein amount didn’t significantly change (**E**), but IL-17B protein significantly increased in psoriasis (**F**). Scale bar, 100 µM. Ctrl *N* = 6; Psor *N* = 10. **, *p* < 0.01. **G**, **H** The secreted protein levels of CXCL8, CCL20, IL-6, IL-1B, and IL-17B in skin fibroblasts (**G**) or skin basal cells (**H**) under TNF-α treatment were determined by ELISA. The protein concentrations were normalized to control group. *N* ≥ 3; *, *p* < 0.05; NS not significant. **i** Flow cytometry histograms of CD80 and CD86 expressions in DCs induced by IL-17B. LPS treatment was used as positive control.

To explore the cell–cell interaction among each cell type of psoriatic skin, we applied ligand-receptor analysis for all upregulated ligand genes and relatively constant expressed receptor genes of each cell type during psoriasis. Full ligand–receptor interactions of psoriatic cell types were visualized in chord plot (Fig. 4A, SI Table 6), The overall interactions uncovered that significantly activated ligands and interactions were from epidermal cells (EpD_Basal2–3, EpD_Spinous), mesenchymal cells (Mes_Fibro, Mes_Peri1–2), and some endothelial cells. Further activity scoring revealed that the top scored ligand–receptor interactions were enriched on these six top ligand secretion resident cell types (EpD_Basal2–3, EpD_Spinous, Mes_Fibro, Mes_Peri1–2) shared a specific receptor immune cell type: type1 dendritic cell (IM_DC1) (Fig. 4B, SI Table 7). Checking the molecules underlying these interactions revealed that the activated ligands of psoriatic epidermal cells include IL-36G, WNT5A, CD58, and so on (SI Table 6), which all had been shown to modulate DCs^39–41^. The activated ligands of psoriatic mesenchymal cells included two main types: ECM related proteins, including SDK1, FGF7, COMP, COL5A3, COL1A1; cytokines or related proteins, including LIF, IL-6, IL-17B, TNFRSF11B, CCL26, CCL19 (Fig. 4C). The inflammatory effects of LIF and IL-6 had been studied in mesenchymal cells^42^, and here we focused on IL-17B, an uncovered inflammatory ligand in mesenchymal cells and an IL-17 family member. We observed IL-17B co-expressed with PDGFRB+ mesenchymal cells in skin dermis layer, and the skin tissue distribution of PDGFRB protein was consistent with the previous studies^43^. Furthermore, we performed immunohischemistry staining for both healthy and psoriatic skin biopsies, and quantitatively compared the expression levels of PDGFRB and IL-17B protein. We found that although the protein amount of PDGFRB in skin dermis didn’t change (Fig. 4E), IL-17B protein was significantly upregulated in psoriatic dermis region (Fig. 4F). We further estimated the cytokine secretion of skin fibroblasts and basal cells under TNF-α stimulation, a canonical signaling involved in psoriasis and indicated by our susceptibility gene loci analysis (Fig. 3C). Upon TNF-α stimulation, CXCL8, CCL20, and IL17-B significantly increased in fibroblasts (Fig. 4G), and the secretion of CCL20, IL-1B were enhanced by basal cells (Fig. 4H). Last, we observed that IL-17B contributed to the surface expression of CD80 and CD86 protein in DCs (Fig. 4I). Thus, these results suggested that the enhanced secretion of IL-17B from dermal mesenchymal cells might actively contribute to the biological functions of immune cells during psoriasis.

## Discussion

**Fig. 5:**
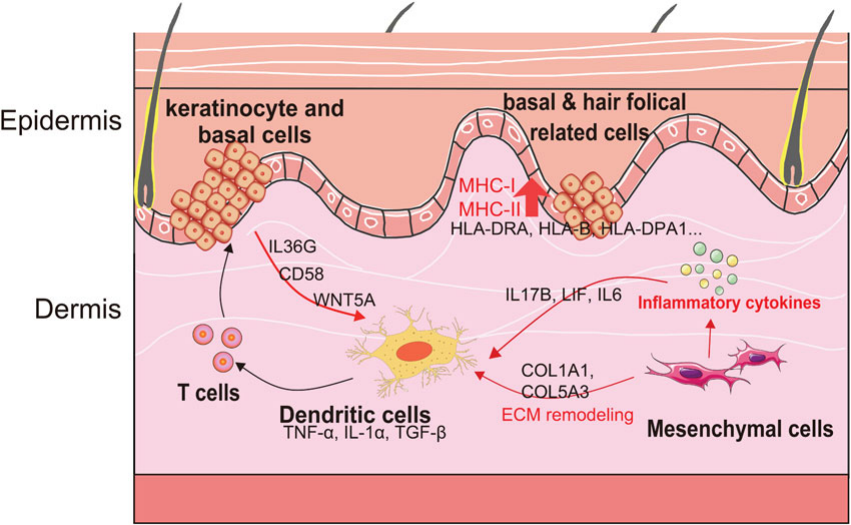
Schematic view of the immune regulation from local resident epidermal cells and mesenchymal cells to DCs. Resident cells including basal cells, hair follicle-like cells, mesenchymal cells potentialize local inflammation by evoking MHC complex genes in psoriatic condition, and these cells can trigger dendritic cells for the inflammatory cascade through remodeling ECM and secreting cytokines such as LIF, IL-6, IL-17B, IL-36, and CD58.

Single cell transcription technology has been used to study epidermal cells and immune cells in psoriatic skin^31,44–46^, but to our knowledge, our study is the first time that comprehensively deconstructs the resident cells from the full-thickness skin of human psoriasis at single cell transcriptional level (Fig. 5). The transcriptional markers of each cell type were mostly in line with previous knowledge (SI Table 2) and mouse skin scRNAseq studies^24,30,31,47^. According to the transcriptional features of our defined human skin cell types, we trained a neural-network learning model to automatically perform cell-type assignment of any skin cells across species. Via this learning model, we further confirmed the evolutional conservation of most skin resident cell types between human and mouse. Furthermore, we found most human melanocytes and SOX10^+^ dermal papilla-like cells, but not human Schwann cells exhibited evolutional specificities. These were probably due to the species difference of the environmental niche, where mouse melanocytes and dermal papilla cells majorly existed near the hair follicle region deeply into the dermis, while human cells usually rested among epidermis/dermis junction^33^.

We mapped the previously identified genomic loci implicated in psoriasis onto specific human skin cell types, and enriched 32 cell type specific genes targeted by the psoriasis genetic predisposition. Surprisingly, although most of these genes were originally interpreted as immune regulators, 24 out of 32 genes were significantly expressed in skin resident cells including keratinocytes or mesenchymal cells. And these 24 genes mainly contributed to IFN signaling and TNF signaling (such as IFNGR2, IL23A, STAT3, STAT5, TNFAIP3). This finding agreed with previous studies of experimental validation^48–51^, and indicated that a decent ratio of genomic loci implicated in psoriasis which could directly affect non-immune cells for immune regulation.

IFN-α could strongly activate immature DCs to produce IL-12, IL-15, IL-18, and IL-23, which acted as upstream cytokines along the IL-23/IL-17 axis to initiate psoriatic inflammation^52^. However, a clinical trial of an anti-IFN-α agent did not show clinical improvement in patients with psoriasis^53^. Our GO analysis showed that upregulated genes of skin resident cell subpopulations in psoriasis vulgaris were abundantly enriched in type I interferon-related functions, which was consistent with the previous result, suggested a crucial role in the disease. However, many immune GO-bioprocesses other than interferon-related functions were enriched from upregulated genes of psoriatic skin resident cells, as shown in (Fig. 3E, F, SI Fig. 3). Importantly, these immune functions could not be dispelled completely by INF-α or frequently-used TNF-α, IL-17, IL-23 targeted therapies. With these enriched alternative immune functions, the upregulated genes in the psoriatic skin resident cells further amplified the inflammatory response, which led to the persistence of localized inflammation, thus caused an alternative hit to patients.

Interestingly, the skin resident cells such as epidermal basal cells, hair follicle-like cells, mesenchymal cells, dermal papilla-like cells, and Schwann cells significantly evoked MHC complex molecules in psoriatic condition. MHC molecules can modulate various types of immune cells via targeting CD4, CD8, CD94 (ref. ^54–56^). And the conditional expression of MHC molecules has been observed in the keratinocytes, dermal fibroblasts, and oligodendrocytes^57–59^. Therefore, our findings that the expression of MHC molecules in psoriatic skin resident cells induced by psoriasis provided a novel insight of the immune regulation from skin resident cells to infiltrated immune cells. Perturbation of MHC related genes has been used for establishing animal model of models of psoriasis^60–63^. The increased expression of MHC-related molecules in human local resident skin cells, especially, HLA-C, may be crucial for transforming the acute inflammation into a chronic disease^64^, which is possibly a key element for building the mouse model of psoriasis.

Ultimately, we identified the upregulated ligand genes of resident epidermal and mesenchymal cells, including many cytokines like LIF, IL-6, IL-17B, IL-36 and CD58. LIF/IL-6 has been identified to sustain the fibroblast production of inflammatory mediators^42^. IL-36 was reported as a molecular linker between keratinocytes and DCs^65^. CD58 was the ligand of CD2 in T cells and NK cells^66^. Here, for the first time, we observed that IL-17B expression increased in the mesenchymal cells in the skin dermis region, possibly upon TNF-α stimulation during psoriasis, which could subsequently trigger DCs activation and potentially other immune cells via binding to its receptor IL17-RB. Therefore, the ligand-receptor linking further demonstrated that local skin resident cells could directly modulate their surrounding immune cells.

In summary, our single cell transcriptional zonation of human full-thickness skin provided a unique insight on the cell-type orchestra in both healthy and psoriatic condition, uncovered the evolutional conserved/specific skin cell types, and systematically proposed the alternative immune-regulatory axis from local resident cells to immune cells. Above all, our findings provided a promising novel target for psoriasis treatment.

## Materials and methods

### Tissue isolation and preparation of single cell suspension

Three patients with definite diagnosis of psoriasis vulgaris were selected as donors, and the full-thickness skins of the lesion were excised surgically. Three healthy donors without systemic diseases undergoing surgery were selected as controls, whose normal skins around the surgical margin were taken for the study. Sample digestion was performed using the full-thickness skin dissociation kit for human (Miltenyi Biotec, Germany). Enzymatic digestion was completed in 2 h, followed by mechanical dissociation using gentleMacs Dissociator (Miltenyi Biotec), running the gentleMACS program h_skin_01. Single cell suspension with the cell counts more than 500 000 and alive cell rate more than 90% observed under a microscope could be tested in the next step.

### Preparation of scRNAseq library, sequencing and data pre-processing

Single cell cDNA libraries were prepared by using 10X Genomics Chromium Single Cell 3’ Reagent Kits (version 3.1) according to the manufacturer’s instruction, and the constructed libraries were sequenced on illumina novaseq 6000 with paired end 150 bp. Raw sequencing data were processed via the 10X Genomics Cell Ranger (version 3.0.2) by using GRCh38.p12 genome assembly. According to the evaluation of quality control, cells harboring <500 transcripts, <1000 unique molecules, >5% ratio of mitochondria, and predicted doublets were removed.

### Cell clustering and marker gene enrichment

Before clustering, we removed the cell cycle genes and obtained the overdispersed genes by estimating the mean and coefficients of variation. The overdispersed genes were then used for cell clustering via Louvain algorithm for community detection (Seurat V2.0 + )^67^. For each main cell type, we further zoomed-in for clustering the sub-clusters. As each cell type with different features, we set the neighboring numbers range between 5–25, and selected PCs according to the elbow plot (normally top 20 PCs). And the resolution was between 0.8–1.5. Some clusters were merged or further separated to eventually obtain significant marker genes for each cluster. The significant marker genes of each cluster were determined via Enrichment Score with a cutoff of false positive rate <0.1(ref. ^68^).

### Probabilistic scoring analysis of cell-type identity in neural network model

To build a classify for scoring the probabilistic cell identity of each cell relative to the defined cell types at the transcriptional level^69,70^, a vanilla neural network model for classification task was constructed in PyTorch framework with Skorch package, and trained the model to interactively learn the transcriptional features of defined cell types. To train the model, we obtained the marker genes of each cell type that previously defined by enrichment score and simultaneously ranked by the expression fold-change. The cross-species alignment was performed as described in the previous study^71^. Data were visualized in the radar-shield plot^72^.

### GO-bioprocess enrichment and ligand-receptor analysis

GO enrichment was performed with custom-built SQL dataset originally from Gene Ontology Resource (www.geneontology.org). Pipeline was modified from goenrich package, highlighting the significant enriched GO terms in red frame and specifically enriching the GO-bioprocess. The multi-layer enrichment plots were visualized via NetworkX. Ligand-receptor analysis were performed via nichenetr package with modification that specifically extract the ligand-to-receptor event, and instead of evaluating the ligand-receptor activity, the ligands were selected via psoriasis upregulated genes of each cell types, and corresponding receptors were extracted with the criteria of constant or upregulated expression. All interactions of each cell type were visualized by chord plot. Ligand-to-receptor score of each cell type-cell type pair was calculated via the ratio between the active interaction event and the total interaction event. The top active interactions were selected once the sorted overall ratio curve of all interactions reaches the edge curve plateaus, thus the cutoff was set as 0.35. Specific ligand-receptor interactions between two cell types were visualized via Sankey diagrams plot.

### Immunofluorescence and immunohistochemistry

For immunohistochemistry staining, skin tissue sections were incubated with primary antibodies of anti-PDGFRB (YT3639, Immunoway, China), anti-IL-17B (bs-2609R, Bioss, China), and secondary antibodies of HRP-Goat anti rabbit IgG (RS0002, Immunoway), HRP-Goat anti mouse IgG (RS0001, Immunoway). For immunofluorescence, skin tissue sections were incubated with primary antibodies of anti-PDGFRB (ab156762, Abcam, UK), anti-IL-17B (bs2609, Bioss), and secondary antibodies of Goat Anti-Rabbit IgG (ab6939, Abcam), Goat Anti Mouse IgG (RS0003, Immunoway). The slides were mounted with Vectashield with DAPI (Vector Laboratories, CA, USA). Laser-scanning confocal images were acquired using the BX51 microscope (Olympus, Japan).

### DC activation

DCs were generated from the bone marrow cells of 8 weeks old female BALB/c mice. The bone marrow cells were centrifuged, washed and cultured in roswell park memorial institute 1640 medium (Hyclone, Utah, USA) containing 10% fetal bovine serum (Sciencell, CA, USA), 1% penicillin/streptomycin (Beyotime), 10 ng/ml recombinant human IL-4 protein (R&D systems, Minnesota, USA) and 20 ng/ml granulocyte macrophage-colony stimulating factor (PeproTech, NJ, USA) and incubated at 37 °C and 5% CO2 for seven days. Cells were cultured with recombinant human IL-17B protein (500 ng/mL) (R&D systems) for 24 h. Cells cultured with medium provided a negative control while cells cultured with LPS (10 ng/mL) (Sigma, Missouri, USA) provided the DC activation positive control. DC phenotypes were analyzed via surface expression of specific markers. Live DCs were immunolabeled with APC-CY7-livedead (Thermo Fisher Scientific, Massachusetts, USA), eFluor450-CD11C (Thermo Fisher Scientific), PE-CD80 (Thermo Fisher Scientific), APC-CD86 (Thermo Fisher Scientific), FITC-MHC II (Thermo Fisher Scientific) at 4 °C for 30 min. Resuspend cells were detected on Beckman Cytoflex LX and the result was analyzed by FlowJo 10.

### PCR-Sequence specific primer of HLA-C*06:02

Fresh blood (200 µl) was collected into EDTA containing tube and DNA was extracted using QIAamp Blood Mini Kit (Qiagen, Germany). PCR was performed according to the manufacturer’s recommendations. Primers of human HLA-C*06:02 were F-TACTACAACCAGAGCGAGGA; R-GGTCGCAGCCATACATCCA. PCR products were electrophoresed in 2% agarose gels containing 0.5 mg/ml ethidium bromide. The gels were run for 35 min at 100 V in TAE buffer, and the gel was scanned.

### Primary cell isolation and ELISA for chemokines detection

Skin samples were placed in 0.25% dispase II (Roche, Switzerland) solution at 4 °C for 16 h. The epidermis and dermis were separated by forceps. The epidermis was cut and put into 0.25% trypsin and 0.02% EDTA digestion (Beyotime), and the cells were cultured in the defined keratinocyte-SFM (Gibco, New York, USA) for 5 days to get primary keratinocytes. The dermis was cut into small pieces and cultured in Dulbecco’s modified eagle medium (Gibco) containing 10% fetal bovine serum (Sciencell), 1% penicillin/streptomycin (Beyotime) for 7 days to get primary fibroblast. Cells were cultured with recombinant human TNF-α protein (Abcam) for 12 h, cells cultured with medium provided a control group. Culture supernatants were collected to measure the levels of cytokines secreted by keratinocytes and fibroblast. The levels of CXCL8 (Raybiotech, Georgia, USA), CCL20 (Abcam), IL-6 (Invitrogen), IL-1B (Abcam), and IL-17B (Novus Biologicals, Colorado, USA) were determined by ELISA according to the manufacturer’s instructions.

### Quantification of images and statistical analysis

Semi-quantification of the staining was performed independently using Image-Pro Plus 6.0. Statistical analysis was performed using GraphPad Prism version 8.0. Data were presented as mean ± SEM. Statistical significance was assessed by Student’s *t*-test.

## Supplementary information

Supplementary Information

Supplementary figure 1

Supplementary figure 2

Supplementary figure 3

Supplementary table 1

Supplementary table 2

Supplementary table 3

Supplementary table 4

Supplementary table 5

Supplementary table 6

Supplementary table 7

## Data Availability

The processed sequencing data reported in this paper is in Gene Expression Omnibus (GEO) database: GSE162183. Raw data in this paper will be deposited in European Genome-Phenome Archive (EGA). Webpage of data visualization: https://yzstudio.one/skin-psoriasis-atlas. Public data used in this study were downloaded from EGA: EGAS00001002927; GEO: GSE130973, GSE150361 & GSE129218. Code details are available upon reasonable request.
