# Supplementary material for: Single cell transcriptional zonation of human psoriasis skin identifies an alternative immunoregulatory axis conducted by skin resident cells
Source: Cell Death Dis. 2021 May 6;12(5):450. doi: 10.1038/s41419-021-03724-6 (PMC8102483; doi:10.1038/s41419-021-03724-6)
Supplement: Supplementary file 1 — Supplementary Information [file 41419_2021_3724_MOESM1_ESM.docx]

**Supplementary Information (SI)**

Supplementary Information summary:

1. SI Figure 1-3

2. SI table 1-7

**Supplementary Figure Legend**

**Figure SI1**

(A) Bar plot visualization of the distribution of total transcript counts of each sequenced cell, red line represents the median transcript number.

(B) Bar plot visualization of the distribution of total unique molecular reads of each sequenced cell, red line represents the median unique molecular reads.

(C) Scatter plot of mitochondrial ratio against total molecular reads in each sequenced cell.

(D) TSNE plot of in silico doublet detection, black dots indicate the estimated doublets

(E) The representation of HLA-C*06:02 allele was performed via PCR-SSP (PCR-sequence specific primer), and visualized as a bend below 250 bp. Only Psor3 expressed HLA-C*06:02 allele.

(F) Violin plot visualization of the expression of marker genes among each cell type, for each cell type, we selected 3 marker genes according to the enrichment score.

(G) Learning curve of the trained neural-network model of our defined skin cell types. The accuracy score was calculated by Sklearn accuracy_score function, indicating the ratio of correct prediction of each epoch. Y axis represents the learning ratio, and x axis represents the epochs.

(H) Violin-swarm plot visualization of the probability percent of our cell-type defined cells against their assigned clusters, according to the trained neural-network model. Each dot represents all cells in that defined cell type, and x axis represents cell types, y axis represents probability score, color indicates the cell type.

(I) Violin plot of the probability percent of the cells with randomized gene expression against our defined cell-types. Red x indicates FDR p-value of 0.01. none of the randomized control group has the score >25%, indicating a successful assignment.

**Figure SI2**

Violin plot visualization of cell-type probabilistic scores of published mouse skin atlas cells in relation to our defined human skin cell types. X axis represents our defined cell types, y axis represents defined cell types from original study. The sub-y axis in each row represents the probabilistic percent of this mouse cell type cells assigning to human cell types.

**Figure SI3**

GO-bioprocess enrichment plot of all up-regulated genes of epidermis cell types and mesenchymal cell types during psoriasis. Red frame highlights the significant enriched GO terms and the selected terminally enriched GO-bioprocess were enlarged at the bottom of each plot.
