# Supplementary figures and images for: Single cell transcriptional zonation of human psoriasis skin identifies an alternative immunoregulatory axis conducted by skin resident cells
Source: Cell Death Dis. 2021 May 6;12(5):450. doi: 10.1038/s41419-021-03724-6 (PMC8102483; doi:10.1038/s41419-021-03724-6)

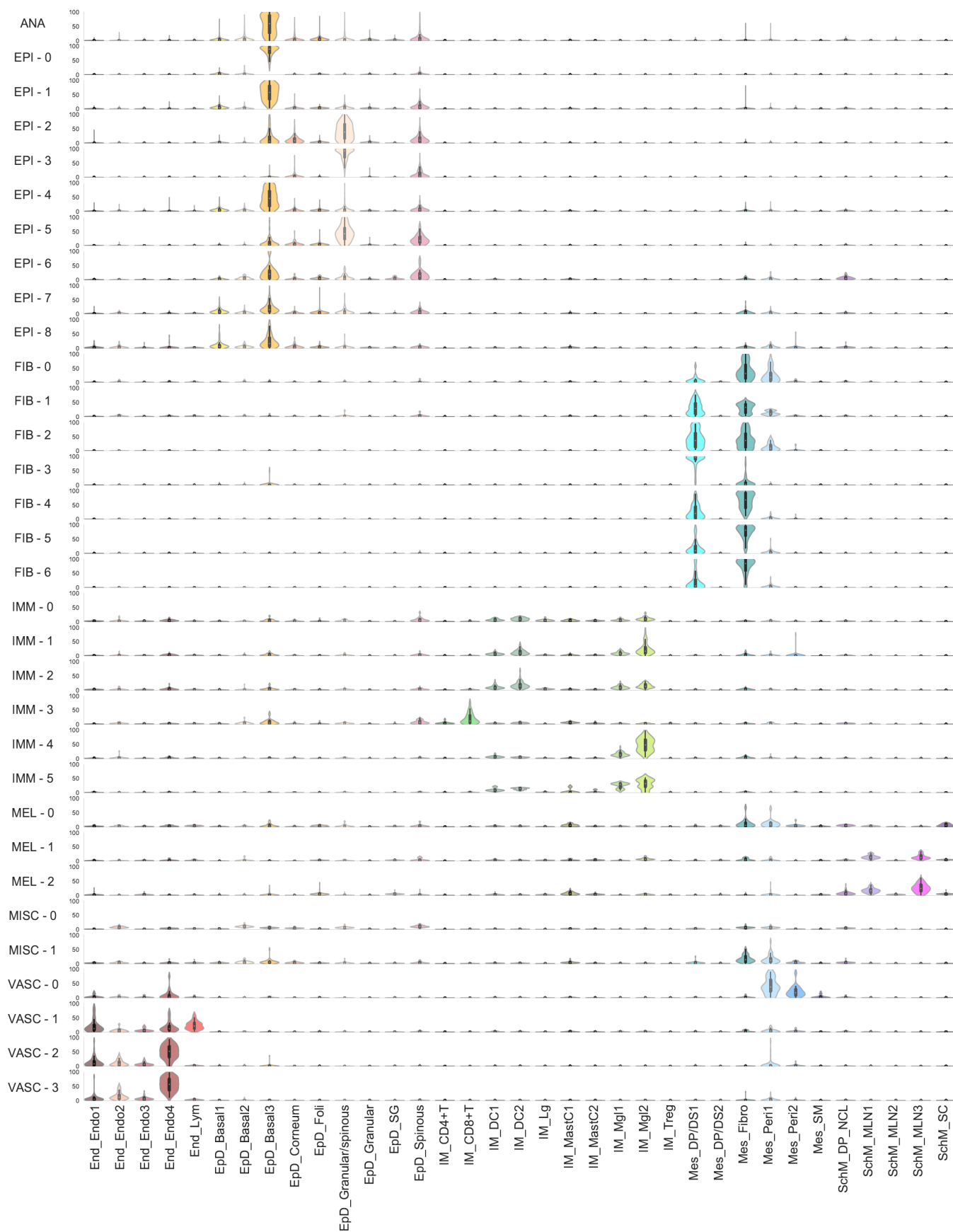

Supplement: Supplementary file 3 — Supplementary figure 2 [file 41419_2021_3724_MOESM3_ESM.pdf]
