# Supplementary material for: Single cell transcriptional zonation of human psoriasis skin identifies an alternative immunoregulatory axis conducted by skin resident cells
Source: Cell Death Dis. 2021 May 6;12(5):450. doi: 10.1038/s41419-021-03724-6 (PMC8102483; doi:10.1038/s41419-021-03724-6)
Supplement: Supplementary file 4 — Supplementary figure 3 [file 41419_2021_3724_MOESM4_ESM.pdf]

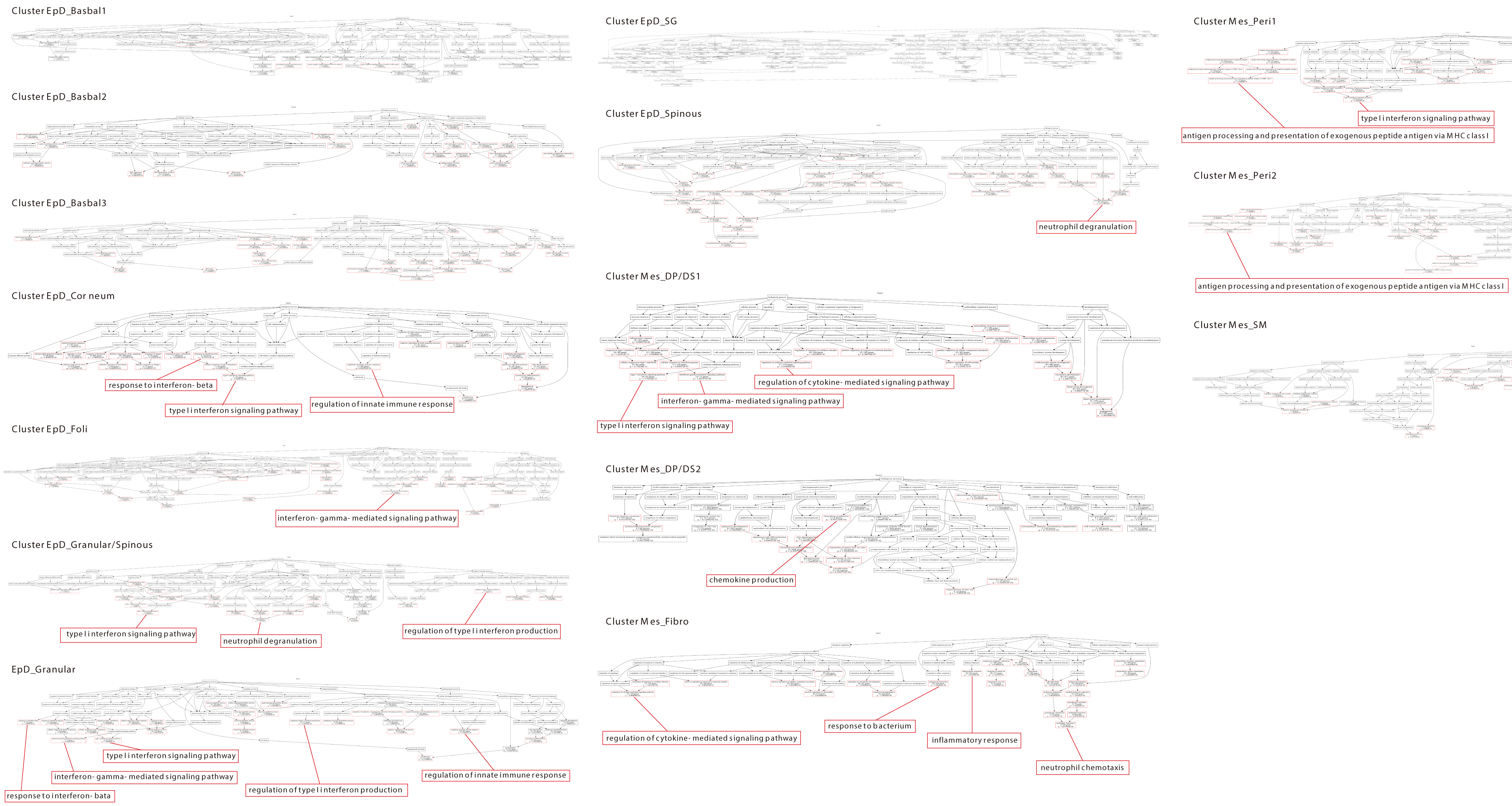

Cluster EpD_Basbal1
Cluster EpD_Basbal2
Cluster EpD_Basbal3
Cluster EpD_Cor neum
response to interferon- beta
type I interferon signaling pathway
regulation of innate immune response
Cluster EpD_Foli
interferon- gamma- mediated signaling pathway
Cluster EpD_Granular/Spinous
type I interferon signaling pathway
neutrophil degranulation
regulation of type I interferon production
EpD_Granular
type I interferon signaling pathway
interferon- gamma- mediated signaling pathway
response to interferon- bata
regulation of type I interferon production
regulation of innate immune response
Cluster EpD_SG
Cluster EpD_Spinous
neutrophil degranulation
Cluster Mes_DP/DS1
regulation of cytokine- mediated signaling pathway
interferon- gamma- mediated signaling pathway
type I interferon signaling pathway
Cluster Mes_DP/DS2
chemokine production
Cluster Mes_Fibro
regulation of cytokine- mediated signaling pathway
response to bacterium
inflammatory response
neutrophil chemotaxis
Cluster Mes_Peri1
type I interferon signaling pathway
antigen processing and presentation of exogenous peptide antigen via MHC class I
Cluster Mes_Peri2
antigen processing and presentation of exogenous peptide antigen via MHC class I
Cluster Mes_SM
